# Effects of foot reflexology massage on pregnant women: a systematic review and meta-analysis of randomized controlled studies

**DOI:** 10.1038/s41598-023-51107-y

**Published:** 2024-01-10

**Authors:** Jia-ming Yang, Ze-qin Li, Hua Ye, Yan-lin Wu, Yi Long, Yan-biao Zhong, Yun Luo, Mao-yuan Wang

**Affiliations:** 1 Department of Rehabilitation Medicine, First Affiliated Hospital of Gannan Medical University, 128 Jinling Road, Zhanggong District, Ganzhou, 341000 Jiangxi China; 2 School of Rehabilitation Medicine, Gannan Medical University, Ganzhou, China; 3 Ganzhou Intelligent Rehabilitation Technology Innovation Center, Ganzhou, China; 4 Ganzhou Key Laboratory of Rehabilitation Medicine, Ganzhou, China

**Keywords:** Psychology, Health care

## Abstract

To explore the effects of foot reflexology massage on anxiety, pain, duration of labor, labor satisfaction, blood pressure, pulse rate and respiratory rate in pregnant women. We systematically searched eight databases for randomized controlled studies on the effects of foot reflexology massage on pregnant women. The inclusion criteria were as follow: participants were pregnant woman; the intervention is foot reflexology or foot massage; the control intervention is placebo, usual care, or no intervention; outcome indicators included pain, anxiety, birth satisfaction, duration of labor, blood pressure, pulse, and respiration; and study type was randomized controlled study. Studies that did not meet the above requirements were excluded. We assessed the quality of the included studies using the Physiotherapy Evidence Database scale, the risk of bias using the Risk of Bias 2.0 tool, and the level of evidence for the outcomes using the Grading of Recommendations Assessment Development and Evaluation. We used Review Manager 5.3 for data analysis and generated funnel plots to assess publication bias. In addition, sensitivity analysis was used to test the stability of the results. A total of 13 randomized controlled studies with 1189 participants were included in this study. Compared to the control group, foot reflexology massage reduced anxiety and pain in pregnant women, shortened the three stages of labor, and increased birth satisfaction. In addition, it also reduced the pulse rate and respiratory rate of pregnant women, but not for blood pressure. Foot reflexology massage can significantly reduce anxiety and pain, shorten the duration of labor, increase birth satisfaction, and stabilize vital signs in pregnant women. It is a safe and non-invasive form of complementary therapy.

PROSPERO registered number: CRD42022359641. URL: https://www.crd.york.ac.uk/prospero/display_record.php?RecordID=359641.

## Introduction

Childbirth is a relatively long process and is accompanied by severe pain^1^. Labor pain is generally considered to be one of the most severe types of pain a woman can experience in her lifetime^2^. This pain exceeded even the maximum pain limits of the Numerical Rating Scale (NRS) and the Visual Analogue Scale (VAS), as most pregnant women indicated a labor pain level of 11 at the time of assessment^3^. Furthermore, pain does not only occur during labor, but can also persist after delivery^4^. Severe pain usually leads to an unpleasant birthing experience, which makes some women fearful and anxious about it^5^. This unpleasant birth experience makes the pregnant women become anxious and depressed, and this negative emotion in turn makes them feel more fearful when facing the next birth^5^. In addition, during childbirth, women are unable to protect their privacy^6^, which inevitably increases their psychological burden, such as anxiety. Moreover, pain causes the sympathetic nerves to become excited^7^, and their nerve endings can release norepinephrine, which causes systemic vasoconstriction leading to increased arterial blood pressure. In addition, sympathetic excitation can positively regulate the myocardium, resulting in a faster heart rate. In terms of breathing, the respiratory rate will increase when the patient is suffering from unbearable pain. Therefore, it is essential to find appropriate ways to reduce pain and anxiety during labor and to maintain stable vital signs in pregnant women.

The main approaches to the management of labor pain and anxiety are pharmacological^8,9^ and non-pharmacological^10–13^. Although the use of drugs for analgesia is more effective, it inevitably has some side effects on the mother and the newborn^8^. Non-pharmacological methods are more convenient and acceptable to women than pharmacological methods, and few adverse effects have been reported. Non-pharmacological methods include music therapy^12,14^, reflexology massage^15,16^, acupuncture^17,18^, relaxation therapy^19^, etc.

In recent years, the interest in foot reflexology massage is increasing year by year in some countries (e.g., Iran, Turkey). There has been a gradual increase in research on the effects of foot reflexology massage on maternal anxiety, pain, duration of labor, labor satisfaction, and vital signs. However, the results varied between studies. The study by Akköz Çevik et al.^20^ showed that foot reflexology significantly reduced maternal anxiety levels, whereas the study by Levy et al.^21^ reached conflicting conclusions. They found that reflexology reduced anxiety levels immediately after treatment compared to the control group; however, one hour after treatment, anxiety levels increased in the experimental group instead^21^. Regarding the duration of labor, two studies found that foot reflexology did not have a significant effect on shortening the first and second stages of labor, but it could significantly shorten the third stage of labor^20,22^. However, the study by Dolatian et al. showed that foot reflexology was effective in shortening all three stages of labor^23^. In addition, there is disagreement between different studies regarding the effects of reflexology on blood pressure, respiratory rate, and pulse rate^24,25^. Therefore, it is necessary to conduct a meta-analysis of relevant studies to form a consistent conclusion. The systematic review and meta-analysis were conducted to explore the effects of foot reflexology massage on anxiety, pain, duration of labor, birth satisfaction, and vital signs in pregnant women.

## Material and methods

Based on the Preferred Reporting Items for Systematic Reviews and Meta-Analyses (PRISMA) guidelines^26,27^, the study has been registered on PROSPERO platform (ID: CRD42022359641).

### Search strategies

We conducted systematic searches of eight databases, including PubMed, Cochrane Library, Scopus, Web of Science, Embase, CNKI, WanFang, and SionMed, with a search deadline of August 20, 2022, languages were limited to English and Chinese. The detailed search strategies for the eight databases are shown in Table S1. In addition, we also searched the references of the included studies to prevent missing relevant literature.

### Study selection

Two authors independently screened the retrieved literature, and any inconsistencies were resolved by a third author. Based on the participants, intervention, comparison, outcomes, and study design (PICOS) principles^28^, we established the inclusion criteria for the study, as shown below.

P: Pregnant woman (both primiparas and multiparas), including both spontaneous labor and artificial labor.

I: The intervention is foot reflexology or foot massage.

C: The control intervention is placebo, usual care, or no intervention.

O: Primary outcomes: pain, anxiety, birth satisfaction, and duration of labor; secondary outcomes: vital signs (e.g., blood pressure, pulse, and respiration).

S: Only randomized controlled studies were included in this study because of the more rational design of randomized controlled trials.

Studies that met the following criteria were excluded: (1) participants were not pregnant women; (2) reflexology massage was not limited to the foot (e.g., shoulder, back, sacral, etc.); (3) studies in which reflexology massage was combined with other intervention modalities such that the effect of reflexology alone could not be shown (e.g., reflexology and Chinese herbal foot bath for the intervention group, and routine care only for the control group); (4) outcomes did not meet the inclusion criteria; (5) study types were non-randomized controls (e.g., semi-experimental studies, quasi-experimental studies, conference abstracts, case reports, reviews, etc.).

### Data extraction

Two authors independently extracted main data from the included studies. Any inconsistencies were decided by a third author who made the final decision.

The data extracted included (1) year of publication; (2) country of study; (3) type of participant; (4) sample size and mean age of participants in each group; (5) time points of outcome measurement; (6) times of intervention; (7) outcomes; and (8) assessment scale (Table 1).

**Table 1:** Baseline characteristics of randomized controlled studies included in this study.

Study	Country	Participants	Groups (sample), age (mean ± SD)	Time points of assessment	Times of intervention	Outcomes	Scale
Akköz Çevik et al. 2021	Turkey	Primiparas	EG: routine nursing/midwifery care and foot reflexology (n = 30), age: unknown; CG: routine nursing/midwifery care (n = 30), age: unknown	Active phase (4–7 cm), transition phase (8–10 cm)	1 session	Pain↓, anxiety↓, birth satisfaction↑, duration of labor↓	VAS, STAI, BSS
Degirmen et al. 2010	Turkey	Pregnant women undergoing cesarean	EG: foot massage (n = 25); CG: no intervention (n = 25); total age: 27.3 ± 4.77 years	Before massage, after massage, 90 min after massage	1 session	Pain↓, DBP, SBP, pulse, respiration↓	NRS
Dolatian et al. 2011	Iran	Primiparas	EG: foot reflexology (n = 40), age: 22.78 ± 3.21 years; CG1: support group (n = 40), age: 22.95 ± 3.57 years; CG2: routine care (n = 40), age: 22.90 ± 3.85 years	Pre-intervention, post-intervention	1 session	Pain↓, duration of labor↓	VAS
Icke et al. 2021	Turkey	Primiparas	EG: foot massage (n = 33), age: 18–35 years; CG: routine procedure (n = 33), age: 18–35 years	Before massage, after massage, 30 min after massage	2 sessions	Pain↓	VAS
Jameei-Moghaddam et al. 2021	Iran	Primiparas and multiparas	EG1: foot reflexology massage on reflex points (n = 32), age: 24.4 ± 6.1 years; EG2: foot reflexology massage on reflex points and each heel (n = 30), age: 25.1 ± 6.5 years; CG: foot reflexology massage on each heel (n = 28), age: 26.0 ± 6.2 years	Pre-intervention, post-intervention	2 sessions	Pain↓, duration of labor	VAS
Kaplan et al. 2021	Turkey	Primiparas	EG: foot reflexology (n = 40), age: 22.2 ± 3.4 years; CG: routine nursing (n = 40), age: 23.6 ± 3.8 years	Pre-application, post-application	1 session	Pain↓, birth satisfaction↑, duration of labor↓	VAS, BSS
Levy et al. 2020	Israel	Primiparas	EG: reflexology and usual care (n = 99), age: 28.6 ± 4.4 years; CG: usual care (n = 90), age: 27.9 ± 4.5 years	0–30 min, 30–90 min	1 session	Anxiety, duration of labor	VAS-A
Moghimi-Hanjani et al. 2015	Iran	Primiparas	EG: routine cares and foot reflexology (n = 40); CG: routine cares and foot massage in other parts (n = 40); total age: 25.56 ± 4.08 years	Before intervention, after intervention	1 session	Anxiety↓, pain↓	STAI, PRI
Navaee et al. 2020	Iran	Primiparas undergoing cesarean	EG: reflexology massage (n = 30), age: 25 ± 4 years; PG: simple massage (n = 30), age: 25 ± 3 years; CG: no intervention (n = 30), age: 26 ± 4 years	Before intervention, after intervention	1 session	Anxiety↓	STAI
Peng et al. 2007	China	Primiparas and multiparas	EG: Chinese medicine foot bath and foot massage (n = 60), age: unknown; CG: Chinese medicine foot bath (n = 60), age: unknown	Pro-intervention, post-intervention	3 sessions	Anxiety↓	SAS
Peng et al. 2008	China	Primiparas and multiparas undergoing cesarean	EG: Chinese medicine foot bath and foot massage (n = 30), age: unknown; CG: Chinese medicine foot bath (n = 30), age: unknown	Pro-intervention, post-intervention	3 sessions	Anxiety↓	SAS
Saatsaz et al. 2016	Iran	Primiparas undergoing cesarean	EG: foot massage and routine cares (n = 52), age: 27.04 ± 2.77 years; CG: routine cares (n = 52), age: 27.75 ± 3.22 years	Before massage, after massage, 90 min after massage	1 session	Pain↓, anxiety, DBP, SBP, pulse↓, respiration↓	VAS, STAI
Sharifi et al. 2022	Iran	Multiparas	EG: foot reflexology (n = 40), age: 29.18 ± 4.93 years; CG: general massage (n = 40), age: 29.10 ± 3.62 years	First to fourth hour after delivery	1 session	Pain↓	VAS

↓ Compared with the control group, outcome indicators decreased after the intervention (P < 0.05). ↑ Compared with the control group, outcome indicators increased after the intervention (P < 0.05).

*BSS* Birth Satisfaction Scale, *CG* control group, *DBP* diastolic blood pressure, *EG* experimental group, *NRS* Numerical Rating Scale, *PEDro* Physiotherapy Evidence Database, *PG* placebo group, *PRI* Pain Rating Index, *SAS* Self­Rating Anxiety Scale, *SBP* systolic blood pressure, *STAI* State-Trait Anxiety Inventory, *VAS* Visual Analogue Scale, *VAS-A* Visual Analogue Scale-Anxiety.

For continuous type data, if there was no significant difference in the baseline comparison between groups, then we extracted the data after the intervention for meta-analysis. If a study provided data for multiple time points on a timeline, data were extracted for each time point. If a study provided data for multiple time points on multiple timelines, then the average data for the same time points were extracted. If a study demonstrated the data before the intervention and the difference before and after the intervention, the data after the intervention was calculated according to Eq. (1). Among them, SD_(a)_ represents the standard deviation (SD) of the endpoint value (after intervention), SD_(b)_ represents the SD of the baseline value (before intervention), and SD_(d)_ represents the SD of the difference. And according to the Cochrane Handbook, R = 0.8 was taken.

1 $$SD_{(a)} = \frac{{2*R*SD_{(b)} + \sqrt {4*R^{2} *SD_{(b)}^{2} - 4*(SD_{(b)}^{2} - SD_{(d)}^{2} )} }}{2}$$

### Quality assessment of included studies

Based on the assessment criteria of the Physiotherapy Evidence Database (PEDro) scale^29^, two authors completed the quality assessment of each study. If there was disagreement between them, the help of a third author was sought to reach agreement. The PEDro scale contains 11 items, which are eligibility criteria and source, random allocation, concealed allocation, baseline comparability, participant blinding, therapist blinding, assessor blinding, adequate follow-up, intention-to-treat analysis, between-group statistical comparisons, and point and variability measurements. Although the scale includes 11 items, only ten items (items 2–11) are scored, with a maximum score of 10. Based on the scores, we classified the methodological quality of the study into four levels: poor (< 4 points), fair (4–5 points), good (6–8 points), and excellent (≥ 9 points).

### Risk of bias

Based on the Cochrane Handbook, two authors independently assessed the risk of bias for the included studies using the Risk of Bias Tool 2.0^30^. Any inconsistent results were resolved after the involvement of a third author. The tool contains five parts, which are the randomization process, deviation from intended intervention, missing outcome data, measurement of the outcomes, and selection of the reported results. The bias for each part was categorized as low risk, some concern, and high risk. Then, an overall bias was derived based on the bias of these five parts. Furthermore, if the number of studies investigating an outcome was sufficiently large, a funnel plot was generated to assess publication bias.

### Grading of evidence quality

According to the Grading of Recommendations Assessment Development and Evaluation (GRADE) criteria^31^, two authors assessed the level of evidence for the outcomes to judge the reliability of the results. The criteria are evaluated for both randomized and observational trials. For randomized trials, the assessment was divided into five panels: risk of bias, inconsistency, indirectness, imprecision, and publication bias. The level of evidence quality was classified into four grades: high, moderate, low, and very low.

### Data synthesis and statistical analyses

Meta-analyses of all data were performed using Review manager version 5.3. For the same outcome, if the same assessment scale was used between studies, the mean difference (MD) was used to estimate the effect value, with 95% confidence intervals (CI) to express the confidence level. If different scales were used to assess the same outcome, the standardized mean difference (SMD) was used to estimate the effect size. Heterogeneity between studies was assessed using the I^2^ statistic, and I^2^ > 50% was considered to be significantly heterogeneous. When the p-value of the heterogeneity test was less than 0.05, a random-effects model was used for the meta-analysis. Conversely, a fixed-effects model was used. Moreover, subgroup analyses were set up to explore whether the results of the effect values were the same under different conditions, sensitivity analysis was used to verify the reliability of the meta-analysis results.

## Results

### Study selection

**Figure 1:**
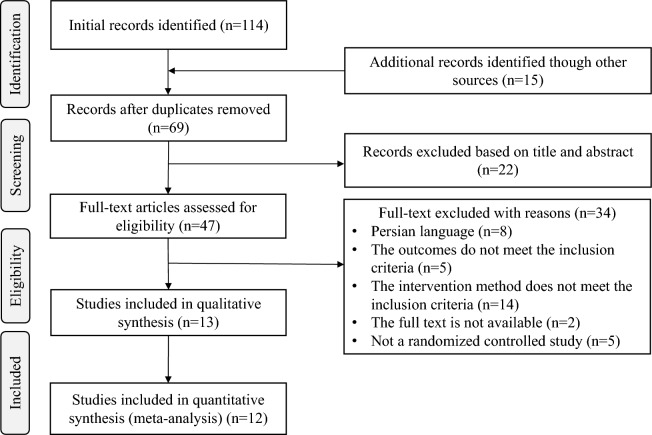
Flow chart of literature screening.

Based on the PRISMA guidelines, we conducted a rigorous screening of the literature, and the flow chart is shown in Fig. 1. A total of 114 records were retrieved from eight databases, and 15 records were obtained from other sources. After removing duplicates, 69 articles remained. After reading the titles and abstracts, 22 articles were eliminated, leaving 47 articles. After reading the full text, 34 articles were excluded, leaving 13 articles. The list of excluded literature and the reasons for exclusion are shown in Table S2. Owing to the absence of tables in the original text of an article^32^, the data could not be extracted. After trying various methods (including sending an email to the article author), the problem was still not resolved. Therefore, 12 studies were finally included in this study for meta-analysis^20–25,33–38^.

### Study characteristics

Of the 13 included studies, 4 studies were from Turkey^20,24,33,38^, 6 studies were from Iran^22,23,25,32,34,35^, 2 studies were from China^36,37^, and 1 study was from Israel^21^. Two studies were Chinese articles^36,37^ and the rest were English articles. Eight studies recruited only primiparas to exclude the effect of multiple deliveries on outcomes^20,21,23,25,32–34,38^, three studies recruited both primiparas and multiparas^22,36,37^, and one study recruited only multiparas^35^. Among them, four studies recruited women who required cesarean delivery^24,25,34,36^. Eight studies used foot reflexology for the intervention^20–23,32–35^ and five studies used foot massage^24,25,36–38^. One study intervened before labor^34^, six studies intervened during labor^20–23,32,33^, and six studies intervened after labor^24,25,35–38^. Two studies conducted three session interventions^36,37^, two studies conducted two sessions interventions^22,38^, and the remaining study had only one session intervention. The specific interventions (e.g., the amount of time each foot received foot reflexology massage) for the experimental and control groups in each study are shown in Table S3. Seven studies assessed pain using the VAS^20,22,23,25,33,35,38^, one studies used the Pain Rating Index (PRI)^32^, and one study used the NRS^24^. Four studies used the State-Trait Anxiety Inventory (STAI) to assess anxiety^20,25,32,34^, two studies used the Self-­Rating Anxiety Scale (SAS)^36,37^, and the other study used the Visual Analogue Scale-Anxiety (VAS-A)^21^. Two studies assessed the birth satisfaction of maternal using the Birth Satisfaction Scale (BSS)^20,33^. The interventionists in each of the above studies were either experienced or had formal training.

### Methodological quality of included studies

We assessed the methodological quality of the included studies using the PEDro, the results of which are shown in Table S4. Three studies used allocation concealment during random assignment^22,33,35^, and one study did not mention comparisons of baseline characteristics between groups^20^. Due to the nature of the intervention, the implementation of the blind method is more difficult. Therefore, only two studies administered blinding to participants^22,35^ and one study administered blinding to assessors^25^; the remaining studies did not mention blinding. Three studies did not have sufficient follow-up numbers (< 85%) and did not use intention-to-treat analysis^35,38^. Consequently, one study scored 8 (quality: good)^22^, two studies scored 7 points (quality: good)^25,33^, eight studies scored 6 points (quality: good)^21,23,24,32,34–37^, one study scored 5 points (quality: fair)^20^, and one study scored 4 points (quality: fair)^38^.

### Risk of bias

**Figure 2:**
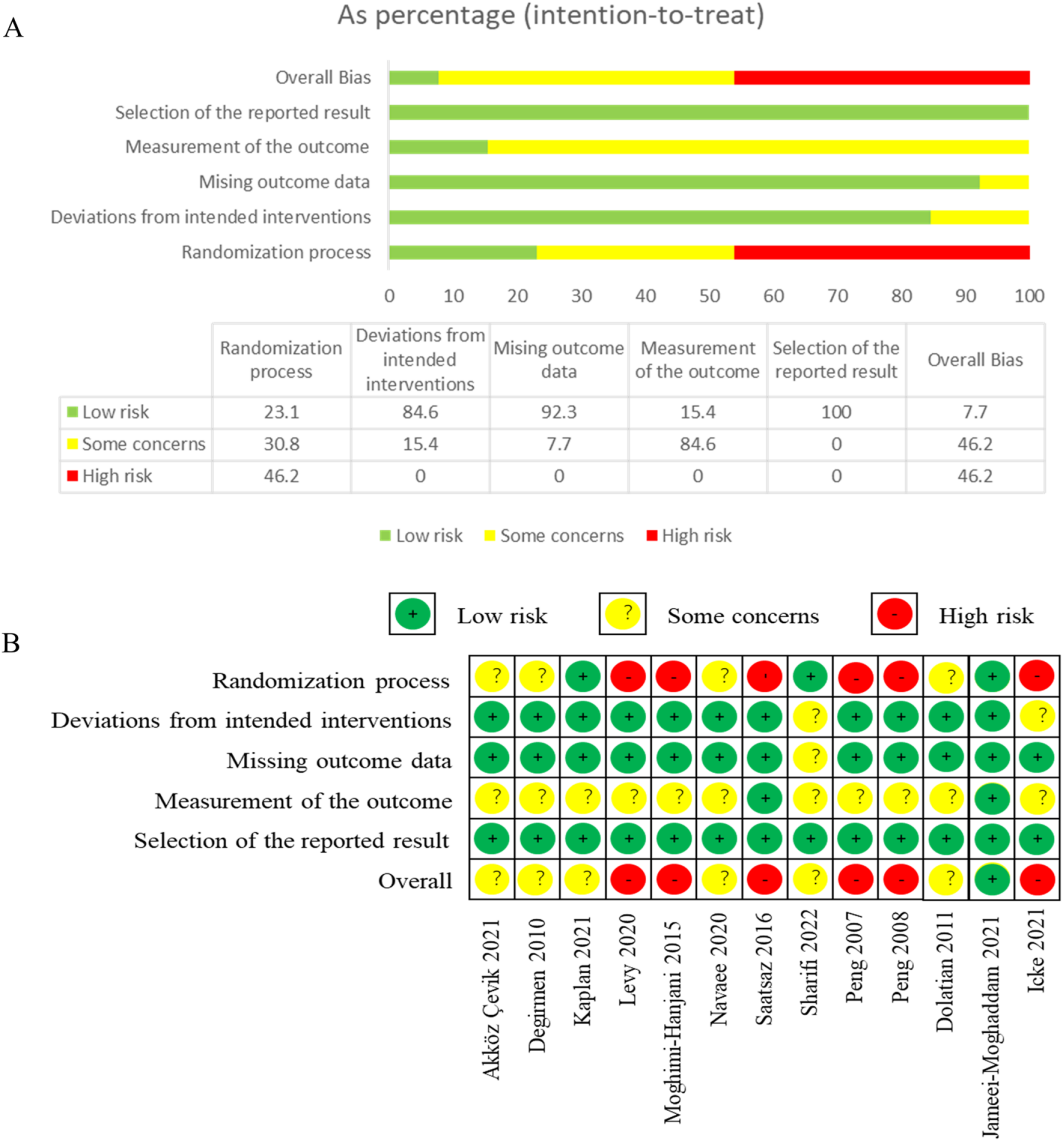
Risk of bias graph and summary of included studies. (**A**) (Risk of bias graph) shows the overall risk of bias in each domain. (**B**) (Risk of bias summary) indicates the risk of bias in each domain for each study.

The risk of bias for the 13 studies is shown in Fig. 2. The randomization process in six studies was rated as high risk, because the method of random sequence generation was problematic and allocation concealment was not performed^21,25,32,36–38^. The randomization process in four studies had some concerns, because there was not enough information to determine whether allocation concealment was performed^20,23,24,34^. The randomization process in three studies used allocation concealment and was therefore rated as low risk^22,33,35^. Some participants in two studies did not complete the trials according to the established intervention protocol, but it may not have a substantial impact on the results, so there is some concern in deviation from the intended intervention^35,38^. In the two studies, participants withdrew for possible health-related reasons (e.g., additional oxytocin) in one study (missing outcome data: some concerns)^35^, and for possible non-health-related reasons (e.g., early withdrawal) in one study (missing outcome data: low risk)^38^. One study was blinded to the assessor^25^ and one study was blinded to the participant and used allocation concealment^22^, so it is unlikely that the assessor and participant had an impact on the outcome assessment (measurement of the outcome: low risk). One study mentioned the use of two scales (VRS and NRS) to assess pain in the methods section, but only the result of NRS scale was presented in the results section for pain intensity levels acquired with VRS were parallel with those of NRS^24^. Therefore, we considered the risk of bias in the study resulting from the selection of reporting results to be low. Consequently, the overall bias was high risk in six studies^21,25,32,36–38^, some concern in six studies^20,23,24,33–35^, and low risk in one study^22^.

### Quality of evidence

We used GRADE profiler software version 3.6.1 to assess the quality of evidence for the outcomes, and the results are shown in Table S5. The quality of evidence for the remaining outcomes, with the exception of birth satisfaction and third stage of labor, was given a downgrade for serious risk of bias, as most studies had a high risk of overall bias. Although the heterogeneity of anxiety, pain, birth satisfaction, respiratory rate, first stage of labor, and second stage of labor was greater than 50%, this heterogeneity was mainly derived from the magnitude of effectiveness between studies rather than from the difference between effectiveness and ineffectiveness. Therefore, the level of evidence for these outcomes was not downgraded by inconsistency. The sample sizes for birth satisfaction, third stage of labor, systolic blood pressure, diastolic blood pressure, pulse rate, and respiratory rate were less than 400, so the level of evidence was downgraded by one level for serious imprecision. The funnel plots of anxiety and first stage of labor were asymmetric, so the level of evidence was downgraded by one level for reporting bias. Consequently, the quality of evidence was moderate for pain, second stage of labor, third stage of labor, and birth satisfaction, and low for anxiety, first stage of labor, systolic blood pressure, diastolic blood pressure, pulse, and respiration.

### Effect of foot reflexology massage on primary outcomes

Six studies investigated the effect of foot reflexology massage on anxiety in pregnant women^20,21,25,34,36,37^, eight studies assessed its effect on pain^20,22–25,33,35,38^, five studies recorded the first and second stage of labor^20–23,33^, three studies recorded the third stage of labor^20,22,23^, and two studies examined its effect on birth satisfaction^20,33^. Since there are three types of scales (STAI, VAS-A and SAS) for assessing anxiety, the SMD was used to estimate the combined effect size. It is important to note that although there are also two types of scales (VAS and NRS) for assessing pain, both are 11-point scales, with 0 representing no pain and 10 representing severe pain, so we still used MD to estimate the combined effect size.

**Figure 3:**
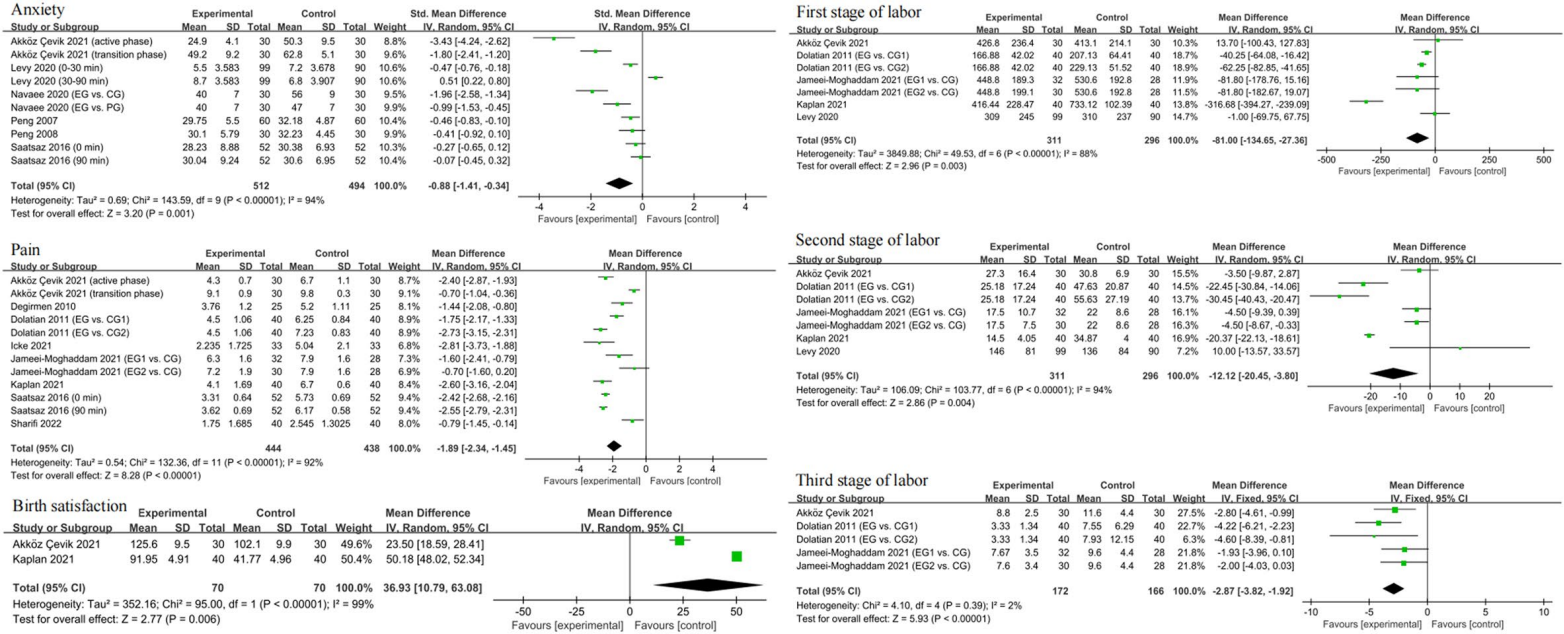
Forest plots of the effects of foot reflexology massage on the primary outcomes.

The forest plot results showed that foot reflexology massage was effective in relieving anxiety (SMD: −0.88, 95% CI: −1.41, −0.34, I^2^ = 94%, P = 0.001), reducing pain (MD: −1.89, 95% CI: −2.34, −1.45, I^2^ = 92%, P < 0.00001), shortening the first stage (MD: −81.00, 95% CI: −134.65, −27.36, I^2^ = 88%, P = 0.003), second stage (MD: −12.12, 95% CI: −20.45, −3.80, I^2^ = 94%, P = 0.004) and third stage (MD: −2.87, 95% CI: −3.82, −1.92, I^2^ = 2%, P < 0.00001) of labor, and increasing birth satisfaction (MD: 36.93, 95% CI: 10.79, 63.08, I^2^ = 99%, P = 0.006) **(**Fig. 3**)**.

### Effect of foot reflexology massage on secondary outcomes

**Figure 4:**
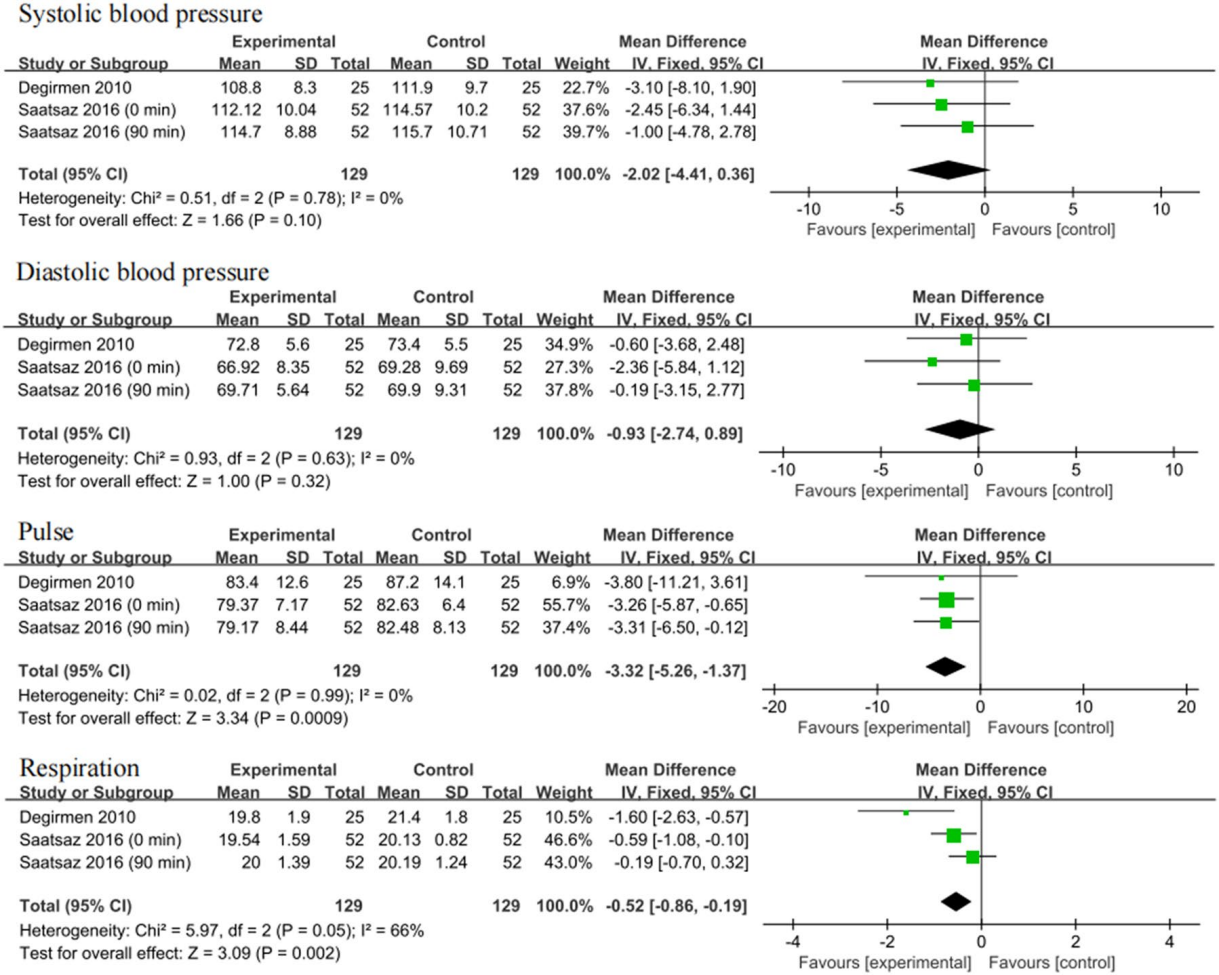
Forest plots of the effects of foot reflexology massage on the second outcomes.

Two studies investigated the effects of foot massage on diastolic blood pressure, systolic blood pressure, pulse and respiration of pregnant women^24,25^. For respiratory rate, although the heterogeneity was relatively high (I^2^ = 66%), P was equal to 0.05, so the fixed-effects model was used. The forest plot results showed that foot massage reduced pulse rate (MD: -3.32, 95% CI: -5.26, -1.37, I^2^ = 0%, P = 0.0009) and respiration rate (MD: -0.52, 95% CI: -0.86, -0.19, I^2^ = 66%, P = 0.002), but there was no significant effect on systolic blood pressure and diastolic blood pressure **(**Fig. 4**)**.

### Subgroup analyses

We performed subgroup analyses according to type of intervention, stage of intervention, times of pregnancies, and mode of childbirth, respectively.

First, we set up subgroup analyses based on the type of intervention. For anxiety, the results were significant for both subgroups. The heterogeneity was very high in the foot reflexology subgroup (96%), while the heterogeneity in the foot massage subgroup was 0%. For pain, the results were significant for both subgroups, but heterogeneity was very high for both (92% and 72%). All eight studies showed that foot reflexology massage reduced maternal pain, only to varying degrees of relief, thus leading to a high degree of heterogeneity.** (**Fig. S1**).**

Second, we set up subgroup analyses based on the stage of intervention. For anxiety, foot reflexology massage can relieve anxiety before labor and after labor, and also has a tendency to reduce anxiety during labor (SMD: −1.25, 95% CI: −2.52, 0.02, I^2^ = 97%, P = 0.05). For pain, foot reflexology massage can reduce pain during labor and after labor** (**Fig. S2**).**

Third, we set up subgroup analyses based on the times of pregnancies. Foot reflexology massage significantly reduced anxiety, pain, first stage of labor, and second stage of labor in both primiparous and multiparous mothers. For primiparous women, the heterogeneity was high for all four outcomes, 95%, 93%, 92%, and 89%, respectively. In contrast, heterogeneity was low for the remaining two subgroups** (**Fig. S3**).**

Fourth, we set up subgroup analyses based on mode of childbirth. Foot reflexology massage significantly reduces anxiety and pain in both natural and cesarean birth. However, the heterogeneity was high in both subgroups** (**Fig. S4**).**

As can be seen from the above four aspects, heterogeneity mainly arises from differences between different primiparous individuals and between different stages of labor for the same individual. Childbirth is a very long process and the pain felt by pregnant women during different stages of labor is usually different. Pain usually affects the anxiety of the pregnant women and hinders the smooth progress of labor, thus prolonging the duration of labor. Moreover, different individuals have different levels of pain tolerance, so this is one of the main sources of heterogeneity.

### sensitivity analyses

In addition, we excluded included studies one by one to assess the stability of anxiety, pain, first stage, second stage and third stage. The results showed that the meta-analyses of anxiety, pain and third stage were stable and highly reliable. However, when the study of Dolatian et al.^23^ was removed, the results of the first stage and second stage of labor became meaningless. In addition, for the first stage of labor, when removing the study of Kaplan et al.^33^ the heterogeneity was reduced to 16%, as well as the funnel plot was evenly distributed on both sides. Therefore, it was not only a source of high heterogeneity in the first stage of labor, but also an important cause of publication bias. Owing to the small number of studies investigating birth satisfaction and vital signs, sensitivity analyses were not performed.

### Publication bias

We examined the potential publication bias by generating funnel plots, and the results are shown in Fig. S5. For anxiety and first stage of labor, the funnel plots were not evenly distributed on either side of the null line, suggesting the possibility of publication bias. For pain and second stage of labor, the funnel plots were evenly distributed on both sides, suggesting little likelihood of publication bias. There were not a sufficient number of studies for the remaining outcome indicators and therefore funnel plot analyses were not performed.

## Discussion

### Principal findings

The results of this systematic review and meta-analysis, which included only randomized controlled studies, showed that foot reflexology massage significantly relieved anxiety and pain, shortened the duration of labor, improved birth satisfaction, and reduced pulse and respiratory rates in pregnant women. However, it had no significant effect on systolic blood pressure and diastolic blood pressure.

### Interpretation

It is important to note that the heterogeneity of most of the outcomes is high, however the main reason for the high heterogeneity is not due to the mixture of negative and positive results, but rather positive results of different magnitudes. Based on the results of the sensitivity analysis, we speculate that the reasons for the high heterogeneity may be the following: (1) The use of scales to assess pain and anxiety is somewhat subjective, which may be influenced by the participant's state (e.g., condition, mood) at the time, such that the scoring results varied widely among individuals. (2) Childbirth is a long process and it is divided into three stages. In different stages, the pain felt by the pregnant women is different. The pain can affect their anxiety and also hinder a smooth delivery. Therefore, the anxiety and pain scores of the same individual may vary during different stages of labor, and the duration of labor can also vary greatly between individuals. It is worth noting that for anxiety and pain, differences due to different scale types may be rather not the main source of their heterogeneity.

It should be noted that the overall bias was high risk in six studies due to an unreasonable randomization process^21,25,32,36–38^. Among the outcome indicators, pain, anxiety and birth satisfaction were subjective indicators, and duration of labor and vital signs were objective indicators. Although there was a high risk of the randomization process in the six studies, the effect of this risk on objective indicators was likely to be small. For subjective indicators, it was difficult for the researchers to assign participants to the experimental group by observing to determine which one had less anxiety and pain. Therefore, the high risk of bias due to the randomization process has an unpredictable impact on the results. Overall, based on the GRADE assessment, we have moderate levels of evidence that foot reflexology massage relieves pain, shortens the second and third stages of labor, and increases birth satisfaction. However, only low levels of evidence suggest that it reduces anxiety, shortens the first stage of labor, and stabilizes vital signs.

Anxiety is a common negative emotion for pregnant women, and pain accompanies almost the entire labor process. The main causes of anxiety in pregnant women are pain, fear for the baby, and worry about something unknown^21^. Anxiety and pain can weaken the contraction activity of the uterus, which can prolong the duration of labor. Furthermore, postpartum pain is a very common occurrence. Studies have shown that postpartum pain increases with the number of deliveries, suggesting that pain is worse in multiparous women than in primiparous women^39^. Therefore, reducing women's labor pain and increasing their birth satisfaction are goals that healthcare workers have been pursuing for a long time.

Reflexology massage is an ancient, non-invasive treatment method, it helps to release enkephalins, which block pain messages to the brain, thus relieving pain and anxiety levels^40^. By stimulating the corresponding reflex zones of the foot^21^ (Fig. S6), the release of oxytocin can be effectively stimulated, thus promoting the contraction of the smooth muscles of the uterus and helping to shorten the duration of labor^20^. Therefore, foot reflexology massage may be very beneficial in facilitating women's delivery. Our study also showed that foot reflexology massage can reduce anxiety (both before and after labor) and pain (both during and after labor) in pregnant women (both primiparas and multiparas). However, current studies focusing on prenatal anxiety in pregnant women are still scarce, suggesting that the focus of future research should be appropriately directed toward the prenatal period.

Our findings are similar to those of Smith et al., who found that massage reduced maternal pain during labor, but had no significant effect on shortening the duration of labor^13^. However, they did not explore the effects of reflexology on maternal labor. A study using a combined intervention of breathing exercises, foot reflexology and massage found that it significantly reduced labor pain and anxiety, shortened the duration of labor and improved vital signs^41^. A recent study showed that foot massage reduced postpartum pain and shortened the second and third stages of labor in women^42^. In conclusion, most of the current evidence suggests that foot reflexology massage can be very helpful in reducing labor pain and anxiety, and enhancing maternal satisfaction with labor^43,44^.

### Strengths and limitations

Strengths: first, we assessed the full range of included studies, including assessment of methodological quality, risk of bias, and level of evidence for outcomes. Second, four subgroups were established to explore the effects of different interventions, different stages of intervention, different times of pregnancies, and different delivery methods on outcomes, to explore the effects of foot reflexology massage on pregnant women from multiple perspectives.

Limitations: first, owing to differences in cultural levels, this study was limited to Chinese and English, which may have led to the omission of some relevant studies in other languages (e.g., Persian). Moreover, because some of the articles were lost, this may have prevented us from analyzing more fully the effects of foot reflexology massage on childbirth, damaging the validity of our work. Second, the specificity of foot reflexology massage made it difficult to blind researchers and participants, which led to a lack of methodological rigor in most of the included studies. Third, the heterogeneity of most outcomes was high, and although it came from varying degrees of positive results, these results still need to be treated with caution. Fourth, both intervention and control groups of the two studies by Peng et al.^36,37^ included Chinese herbal footbaths, which may overestimate the efficacy of foot massage for anxiety and should be treated with caution.

### Clinical implications

Pregnancy and childbirth are two major events in a woman's life, and many women face childbirth every year. Labor pains usually accompany the entire labor process and even continue after delivery. On the one hand, pain is one of the main sources of anxiety in pregnant women. On the other hand, pain can also hinder the smooth progress of labor and thus prolong the duration of labor. Therefore, it has been a long-standing pursuit of obstetricians and gynecologists to find safe and effective ways to reduce pain and anxiety and enhance the well-being of pregnant women. This study showed that foot reflexology massage was effective in reducing pregnant women's anxiety and pain, shortening the duration of labor, and increasing birth satisfaction. Moreover, few studies on the side effects of foot reflexology massage have been reported, indicating that it is a safe and reliable treatment method. Therefore, we recommend foot reflexology massage as a routine clinical care method for prenatal and postpartum to enhance women's well-being. Regarding the duration of foot reflexology massage, according to Table S3, about 20 min of reflexology massage per foot is helpful.

### Research implications

This study synthesizes current randomized controlled studies published in English and Chinese on the effects of foot reflexology massage on pregnant women, providing more comprehensive and more credible evidence for the positive effects of foot reflexology massage on pregnant women. However, the current studies generally have some problems, such as unreasonable randomization process and limited use of blind method, which have led to limited study quality. Therefore, future studies need to be rationally designed in terms of both the randomization process and the blinding method to provide higher quality studies.

## Conclusion

The results of this study showed that foot reflexology massage is effective for pregnant women in relieving anxiety, reducing pain, shortening all the three stages of labor, and regulating respiration and pulse to stabilize their vital signs. Therefore, we are recommending the use of foot reflexology massage for prenatal and postnatal clinical care to promote the birthing experience for pregnant women.

### Supplementary Information

Supplementary Information.

## Data Availability

All data for this study are included in the manuscript and supplementary materials.
