# Supplementary material for: Effects of foot reflexology massage on pregnant women: a systematic review and meta-analysis of randomized controlled studies
Source: Sci Rep. 2024 Jan 10;14:1012. doi: 10.1038/s41598-023-51107-y (PMC10781947; doi:10.1038/s41598-023-51107-y)
Supplement: Supplementary file 1 — Supplementary Information. [file 41598_2023_51107_MOESM1_ESM.pdf]

Supplementary materials for *Effects of foot reflexology massage on pregnant women: A systematic review and meta-analysis of randomized controlled studies*.

Jia-ming Yang; Ze-qin Li; Hua Ye; Yan-lin Wu; Yi Long; Yan-biao Zhong; Yun Luo; Mao-yuan Wang

Table S1. The search strategy of PubMed database.

Table S2. List of excluded literature and reasons for exclusion (34 studies).

Table S3. The specific intervention protocols for the experimental and control groups of each study.

Table S4. The Physiotherapy Evidence Database (PEDro) score of each study.

Table S5. GRADE evidence profiles for primary outcomes and secondary outcomes among the trials included in the systematic review.

Figure S1. Subgroup analyses for anxiety and pain, according to the type of intervention.

Figure S2. Subgroup analyses for anxiety and pain, according to the stage of intervention.

Figure S3. Subgroup analyses for anxiety, pain, first stage of labor, and second stage of labor, according to the number of pregnancies.

Figure S4. Subgroup analyses for anxiety and pain, according to the mode of delivery.

Figure S5. Funnel plots of anxiety, pain, first stage of labor and second stage of labor.

Table S1. The search strategy of each database.

**PubMed**	
#1	foot reflexology OR foot massage
#2	pregnant woman OR primigravida woman OR expectant mother
#3	puerpera OR lying-in woman OR unipara OR primipara OR primiparous OR cesarean
#4	anxiety OR angst OR social anxiety OR anxiousness
#5	pain OR ache
#6	randomized controlled OR RCT OR controlled trial OR clinical trial
#7	#2 OR #3
#8	#4 OR #5
#9	#1 AND #6 AND #7 AND #8
**Web of Science/ Scopus**	
#1	"foot reflexology" OR "foot massage"
#2	"pregnant woman" OR "primigravida woman" OR "expectant mother" OR "puerpera" OR "lying-in woman" OR "unipara" OR "primipara" OR "primiparous" OR "cesarean"
#3	"anxiety" OR "angst" OR "social anxiety" OR "anxiousness" OR "depressive" OR "depression" OR "depressive disorder" OR "depressive neurosis" OR "endogenous depression" OR "depressive syndrome" OR "neurotic depression" OR "melancholia" OR "unipolar depression" OR "pain" OR "ache"
#4	"randomized controlled" OR "RCT" OR "controlled trial" OR "clinical trial"
#5	#1 AND #2 AND #3 AND #4

Embase	
#1	‘foot reflexology’:ti,ab,kw OR ‘foot massage’:ti,ab,kw
#2	‘pregnant woman’:ti,ab,kw OR ‘primigravida woman’:ti,ab,kw OR ‘expectant mother’:ti,ab,kw
#3	‘puerpera’:ti,ab,kw OR ‘lying-in woman’:ti,ab,kw OR ‘unipara’:ti,ab,kw OR ‘primipara’:ti,ab,kw OR ‘primiparous’:ti,ab,kw OR ‘cesarean’:ti,ab,kw
#4	‘anxiety’:ti,ab,kw OR ‘angst’:ti,ab,kw OR ‘social anxiety’:ti,ab,kw OR ‘anxiousness’:ti,ab,kw
#5	‘depressive’:ti,ab,kw OR ‘depression’:ti,ab,kw OR ‘depressive disorder’:ti,ab,kw OR ‘depressive neurosis’:ti,ab,kw OR ‘endogenous depression’:ti,ab,kw OR ‘depressive syndrome’:ti,ab,kw OR ‘neurotic depression’:ti,ab,kw OR ‘melancholia’:ti,ab,kw OR ‘unipolar depression’:ti,ab,kw
#6	‘pain’:ti,ab,kw OR ‘ache’:ti,ab,kw
#7	‘randomized controlled’:ti,ab,kw OR ‘RCT’:ti,ab,kw OR ‘controlled trial’:ti,ab,kw OR ‘clinical trial’:ti,ab,kw
#8	#2 OR #3
#9	#4 OR #5 OR #6
#10	#1 AND #7 AND #8 AND #9
**Cochrane Library**	
#1	‘foot reflexology’:ti,ab,kw OR ‘foot massage’:ti,ab,kw
#2	‘pregnant woman’:ti,ab,kw OR ‘primigravida woman’:ti,ab,kw OR ‘expectant mother’:ti,ab,kw
#3	‘puerpera’:ti,ab,kw OR ‘unipara’:ti,ab,kw OR ‘primipara’:ti,ab,kw OR ‘primiparous’:ti,ab,kw OR ‘cesarean’:ti,ab,kw

#4	‘anxiety’:ti,ab,kw OR ‘angst’:ti,ab,kw OR ‘social anxiety’:ti,ab,kw OR ‘anxiousness’:ti,ab,kw
#5	‘depressive’:ti,ab,kw OR ‘depression’:ti,ab,kw OR ‘depressive disorder’:ti,ab,kw OR ‘depressive neurosis’:ti,ab,kw OR ‘endogenous depression’:ti,ab,kw OR ‘depressive syndrome’:ti,ab,kw OR ‘neurotic depression’:ti,ab,kw OR ‘melancholia’:ti,ab,kw OR ‘unipolar depression’:ti,ab,kw
#6	‘pain’:ti,ab,kw OR ‘ache’:ti,ab,kw
#7	‘randomized controlled’:ti,ab,kw OR ‘RCT’:ti,ab,kw OR ‘controlled trial’:ti,ab,kw OR ‘clinical trial’:ti,ab,kw
#8	#2 OR #3
#9	#4 OR #5 OR #6
#10	#1 AND #7 AND #8 AND #9
**SionMed/ CNKI/WanFang**	
#1	足底反射疗法 OR 足底按摩
#2	抑郁 OR 焦虑 OR 情志异常 OR 抑郁症状 OR 抑郁情绪 OR 疼痛
#3	随机对照 OR 随机分组 OR 随机 OR RCT
#4	孕妇 OR 产妇 OR 剖宫产
#5	#1 AND #2 AND #3 AND #4

Table S2. List of excluded literature and reasons for exclusion (34 studies).

**Study**	**Title**	**Reason**
Abasi et al. 2009	Study of the effect of massage therapy on the intensity of labor	The outcomes do not meet the inclusion criteria
Abbaspoor et al. 2014	Effect of foot and hand massage in post-cesarean section pain control: a randomized control trial	The intervention does not meet the inclusion criteria
Aksu et al. 2021	The Effect of Reflexology on Lactation in Women Who Had Cesarean Section: A Randomized Controlled Pilot Study	The outcomes do not meet the inclusion criteria
Baljon et al. 2022	Effectiveness of Breathing Exercises, Foot Reflexology and Massage (BRM) on Maternal and Newborn Outcomes Among Primigravidae in Saudi Arabia: a Randomized Controlled Trial	The intervention does not meet the inclusion criteria
Bolbol-Haghighi et al. 2016	Effect of massage therapy on duration of labour: A randomized controlled trial	The intervention does not meet the inclusion criteria
Çankaya et al. 2020	The Effect of Reflexology on Lactation and Postpartum Comfort in	The outcomes do not meet the inclusion

	Caesarean-Delivery Primiparous Mothers: A Randomized Controlled Study	criteria
Chang et al. 2002	Effects of massage on pain and anxiety during labour: a randomized controlled trial in Taiwan	The intervention does not meet the inclusion criteria
Field et al. 1997	Labor pain is reduced by massage therapy	The intervention does not meet the inclusion criteria
Hamideh Mortazavi et al. 2012	Effects of massage therapy and presence of attendant on pain, anxiety and satisfaction during labor	The intervention does not meet the inclusion criteria
Hanjani et al. 2012	Effect of foot reflexology on pain intensity and duration of labor on primiparous	Persian language
Hassani et al. 2015	The effect of foot reflexology on physiologic indices and pain severity following cesarean delivery	Not a randomized controlled study
Irani et al. 2015	The effect of hand and foot massage on pain and anxiety	The intervention does not meet the inclusion criteria

Janssen et al. 2012	Massage therapy and labor outcomes: a randomized controlled trial	The intervention does not meet the inclusion criteria
Jenabi et al. 2012	The effect of reflexology on relieving the labor pain	Persian language
Karami et al. 2007	Effect of massage therapy on severity of pain and outcome of labor in primipara	The intervention does not meet the inclusion criteria
Khloobagheri et al. 2020	Effect of foot reflexology with auriculotherapy on pain after elective cesarean section: a randomized clinical trial	The intervention does not meet the inclusion criteria
Khoshtarash et al. 2012	Effects of foot reflexology on pain and physiological parameters after cesarean section	Persian language
Liu et al. 2006	足底按摩减轻剖宫产术后疼痛的探讨 [Exploration of foot massage to reduce post-operative pain after cesarean delivery]	Not a randomized controlled study
Mehdizadeh Tourzani et al. 2013	The Effect of foot reflexology on anxiety during of labor on primiparous	Persian language
Mirhosseini et al.	Effect of aromatherapy massage by orange essential oil on post-cesarean	The intervention does not meet the

2021	anxiety: a randomized clinical trial	inclusion criteria
Mirzaei et al. 2010	Effect of foot reflexology on duration of labor and severity of first-stage labor pain	The full text is not available
Moghimi Hanjani et al. 2013	The effect of foot reflexology on pain intensity and duration of labor on primiparous	Persian language
Mohammadifard et al. 2022	The effect of foot reflexology and H7 point acupressure on anxiety in women undergoing amniocentesis	The intervention does not meet the inclusion criteria
Mokhtari et al. 2010	Comparison of impact of foot reflexology massage and Bensone relaxation on severity of pain after cesarean section: a randomized trial	Persian language
Razmjoo et al. 2012	Effect of foot reflexology on pain and anxiety in women following elective cesarean section	Persian language
Rezaei et al. 2017	Massage-therapy and post cesarean pain control	Persian language
Şanli et al. 2022	Effect of Foot Massage on Labor Pain in Parturient Women	The full text is not available
Sharma et al. 2019	Study to Assess the Effectiveness of Foot and Hand Massage on Reducing	The intervention does not meet the

	Pain among Post Natal Mothers Who Had Undergone Caesarean Section	inclusion criteria
Shen et al. 2020	足底反射疗法对护理剖宫产产妇泌乳及产后舒适度的影响研究 [Effect of foot reflexology on lactation and postpartum comfort in nursing cesarean section mothers]	The outcomes do not meet the inclusion criteria
Taghinejad et al. 2012	Comparison between massage and music therapies to relieve the severity of labor pain	The intervention does not meet the inclusion criteria
Valiani et al. 2022	The effect of foot reflexology on duration of labor and neonatal apgar score in nulliparous women: A randomized clinical trial	The outcomes do not meet the inclusion criteria
Valiani et al. 2010	Reviewing the effect of reflexology on the pain and certain features and outcomes of the labor on the primiparous women	Not a randomized controlled study
Xue et al. 2016	Postoperative Foot Massage for Patients after Caesarean Delivery	Not a randomized controlled study
Yılar Erkek et al. 2018	The Effect of Foot Reflexology on the Anxiety Levels of Women in Labor	Not a randomized controlled study

Table S3. The specific intervention protocols for the experimental and control groups of each study.

**Study**	**Experimental group**	**Control group**
Akköz Çevik et al. 2021	Participants received routine care and a 10-minute (5 minutes per foot) massage followed by 40 minutes (20 minutes per foot) of foot reflexology.	Participants received only routine treatment, care, and practices of the hospital.
Degirmen et al. 2010	Participants received a 5-minute massage on each foot for a total of 10 minutes.	Participants did not receive massage interventions.
Dolatian et al. 2011	Participants received one session of foot reflexology for a total of 40 minutes (20 minutes per foot).	Support group: participants received spiritual, emotional and verbal support from the researcher for 40 minutes.<br>Routine care group: participants received only routine care.
Icke et al. 2021	Participants received 2 sessions of massage for 20 minutes each (10 minutes per foot).	Participants received care for routine procedures.

Jameei-Moghaddam et al. 2021	Intervention group 1: two 30-min sessions of massage therapy on reflex points of each sole.<br>Intervention group 2: one 30-min session of massage therapy on reflex points and one 30-min session of massage therapy on each heel.	Participants received two 30-min sessions of massage therapy on each heel.
Kaplan et al. 2021	Participants received 30 minutes of foot reflexology (15 minutes per foot).	Participants received care for routine procedures.
Levy et al. 2020	Participants received conventional anxiety treatment and 30 minutes of foot reflexology.	Participants received conventional anxiety treatment.
Moghimi-Hanjani et al. 2015	Participants received routine care and 40 minutes of foot reflexology (20 minutes per foot).	Participants received routine care and massage in other parts of the foot.
Navaee et al. 2020	Participants began with a 10-minute massage of both feet, followed by 15 minutes of pressure in the reflex zones, and finally a 5-minute massage of all parts of the feet soles (30 minutes total).	Simple massage group: all the reflex points of the foot (except for anxiety points) were massaged for 30 min with less pressure than in the reflex massage

		group.<br>Control group: participants did not receive any intervention and only completed the questionnaire after 30 min.
Saatsaz et al. 2016	Participants received a routine treatment and a 10-minute foot massage (5 minutes per foot).	Participants received only routine care.
Sharifi et al. 2022	Participants underwent a 4-minute general massage on each foot, followed by a 2-minute specific reflex massage on each of the three acupuncture points of the uterus, pituitary and solar plexus in the form of rotational pressures.	Participants received general massage on each foot for 4 min, followed by rotational pressures on a neutral point on the lateral side of the heel (placebo point) for 6 minutes.
Peng et al. 2007	Participants received a massage while taking a Chinese herbal footbath, with 3 interventions of 10 minutes each.	Participants performed only Chinese herbal footbaths, with 3 interventions of 10 minutes each.
Peng et al. 2008	Participants received a massage while taking a Chinese herbal footbath, with 3 interventions of 10 minutes each.	Participants performed only Chinese herbal footbaths, with 3 interventions of 10 minutes each.

Table S4. The Physiotherapy Evidence Database (PEDro) score of each study.

**Study**	**(1)**	**(2)**	**(3)**	**(4)**	**(5)**	**(6)**	**(7)**	**(8)**	**(9)**	**(10)**	**(11)**	**Score**	**Study Quality**
Akköz Çevik et al. 2021	Yes	1	0	0	0	0	0	1	1	1	1	5	Fair
Degirmen et al. 2010	Yes	1	0	1	0	0	0	1	1	1	1	6	Good
Dolatian et al. 2011	Yes	1	0	1	0	0	0	1	1	1	1	6	Good
Icke et al. 2021	Yes	1	0	1	0	0	0	0	0	1	1	4	Fair
Jameei-Moghaddam et al. 2021	Yes	1	1	1	1	0	0	1	1	1	1	8	Good
Kaplan et al. 2021	Yes	1	1	1	0	0	0	1	1	1	1	7	Good
Levy et al. 2020	Yes	1	0	1	0	0	0	1	1	1	1	6	Good
Moghimi-Hanjani et al. 2015	Yes	1	0	1	0	0	0	1	1	1	1	6	Good
Navaee et al. 2020	Yes	1	0	1	0	0	0	1	1	1	1	6	Good
Peng et al. 2007	Yes	1	0	1	0	0	0	1	1	1	1	6	Good
Peng et al. 2008	Yes	1	0	1	0	0	0	1	1	1	1	6	Good
Saatsaz et al. 2016	Yes	1	0	1	0	0	1	1	1	1	1	7	Good

Sharifi et al. 2022	Yes	1	1	1	1	0	0	0	0	1	1	6	Good

Notes: Yes, one point; No, score 0. A total PEDro score is achieved by adding the ratings of (2) to (11) for a combined total score between 0 to 10.

(1) eligibility criteria were specified, (2) random allocation, (3) concealed allocation, (4) baseline comparability, (5) participant blinding, (6) therapist blinding, (7) assessor blinding, (8) adequate follow-up (> 85%), (9) intention-to-treat analysis, (10) between-group statistical comparisons, and (11) point and variability measurements.

Table S5. GRADE evidence profiles for primary outcomes and secondary outcomes among the trials included in the systematic review.

No. of studies	Design	Risk of bias	Inconsistency	Indirectness	Imprecision	Other considerations	EG (n)	CG (n)	Absolute effect	Quality	Importance
Anxiety (follow-up 1~3 sessions; measured with: STAI, SAS and VAS-A; Better indicated by lower values)											
6	Randomized trials	Serious[1]	No[2]	No	No	Reporting bias[3]	512	494	SMD 0.88 lower (1.41 to 0.34 lower)	++−− Low	Critical
Pain (follow-up 1~2 sessions; measured with: VAS and NRS; Better indicated by lower values)											
8	Randomized trials	Serious[1]	No[2]	No	No	None	444	438	MD 1.89 lower (2.34 to 1.45 lower)	+++− Moderate	Critical
Birth satisfaction (follow-up mean 1 session; measured with: BSS; Better indicated by higher values)											
2	Randomized trials	No	No[2]	No	Serious[4]	None	70	70	MD 36.93 higher (10.79 to 63.08 higher)	+++− Moderate	Critical
First stage of labor (follow-up 1~2 sessions; Better indicated by lower values)											

5	Randomized trials	Serious[5]	No[2]	No	No	Reporting bias[3]	311	296	MD 81 lower (134.65 to 27.36 lower)	++−− Low	Critical
Second stage of labor (follow-up 1~2 sessions; Better indicated by lower values)											
5	Randomized trials	Serious[5]	No[2]	No	No	None	311	296	MD 12.12 lower (20.45 to 3.80 lower)	+++− Moderate	Critical
Third stage of labor (follow-up 1~2 sessions; Better indicated by lower values)											
3	Randomized trials	No	No	No	Serious[4]	None	172	166	MD 2.87 lower (3.82 to 1.92 lower)	+++− Moderate	Critical
Systolic blood pressure (follow-up mean 1 session; Better indicated by lower values)											
2	Randomized trials	Serious[6]	No	No	Serious[4]	None	129	129	MD 2.02 lower (4.41 lower to 0.36 higher)	++−− Low	Important

Diastolic blood pressure (follow-up mean 1 session; Better indicated by lower values)										
2	Randomized trials	Serious[6]	No	No	Serious[4]	None	129	129	MD 0.93 lower (2.74 lower to 0.89 higher)	++−− Low	Important
Pulse rate (follow-up mean 1 session; Better indicated by lower values)										
2	Randomized trials	Serious[6]	No	No	Serious[4]	None	129	129	MD 3.32 lower (5.26 to 1.37 lower)	++−− Low	Important
Respiration rate (follow-up mean 1 session; Better indicated by lower values)										
2	Randomized trials	Serious[6]	No[2]	No	Serious[4]	None	129	129	MD 0.52 lower (0.86 to 0.19 lower)	++−− Low	Important

[1] The overall bias for three studies is high risk.

[2] Although that heterogeneity is high, the heterogeneity is caused by differences in the magnitude of effectiveness rather than by effectiveness and ineffectiveness.

[3] The funnel plot is not symmetrical.

[4] The total sample size is less than 400.

[5] The overall bias for one study is high risk and for three studies is some concerns.

[6] The overall bias in the two studies is high risk.

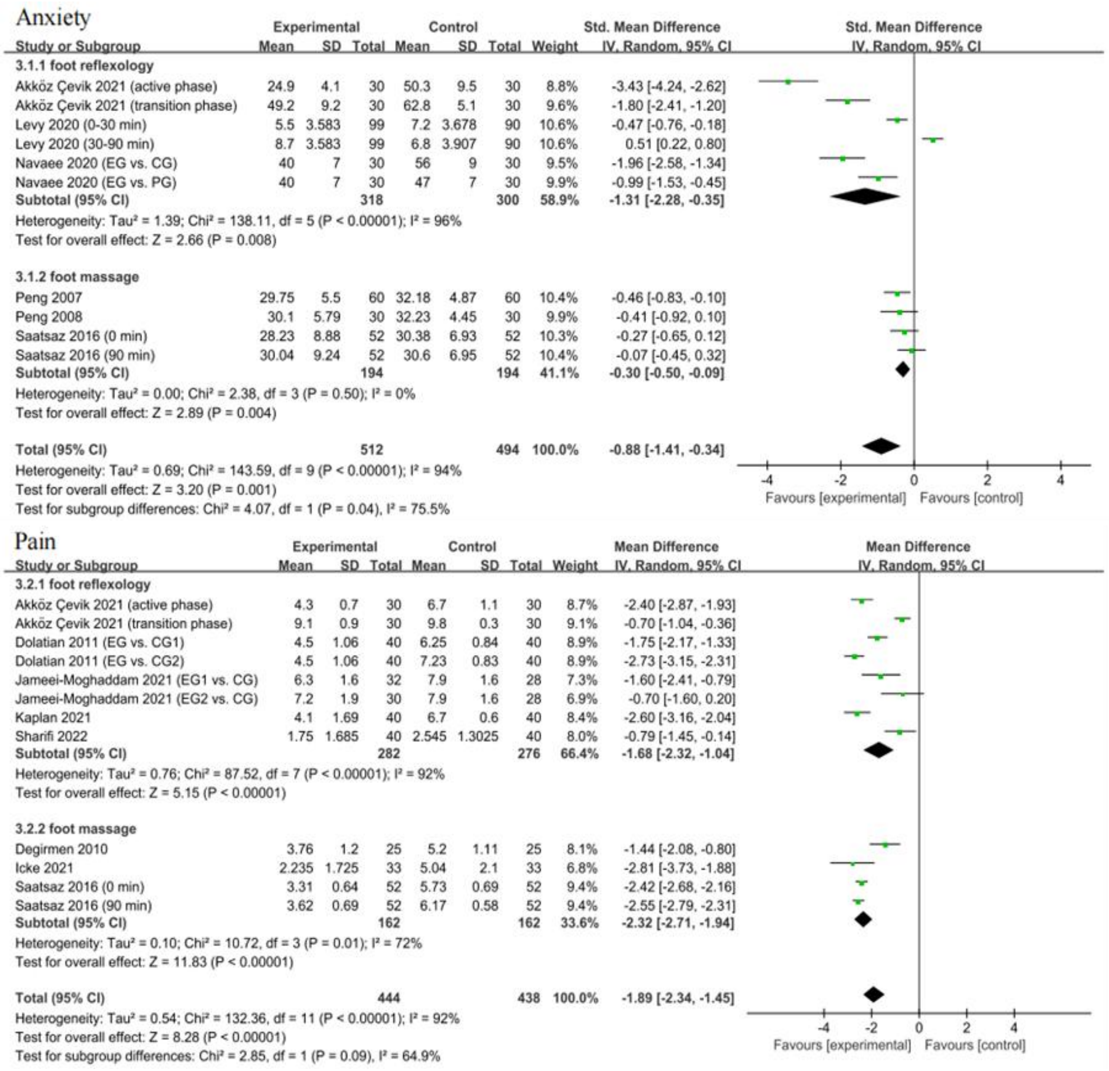

Figure S1. Subgroup analyses for anxiety and pain, according to the type of intervention.

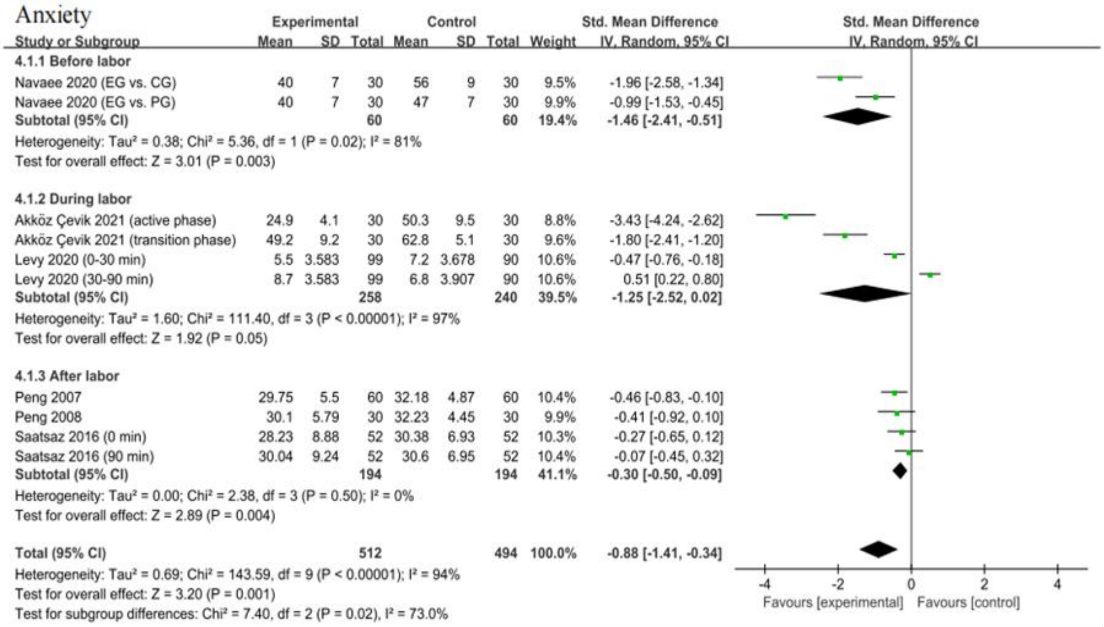

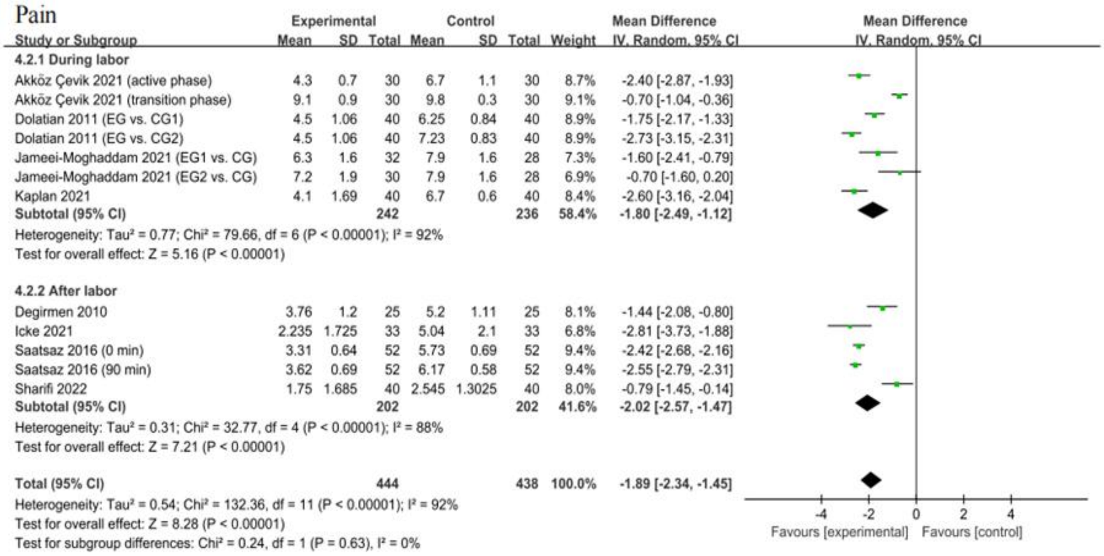

Figure S2. Subgroup analyses for anxiety and pain, according to the stage of intervention.

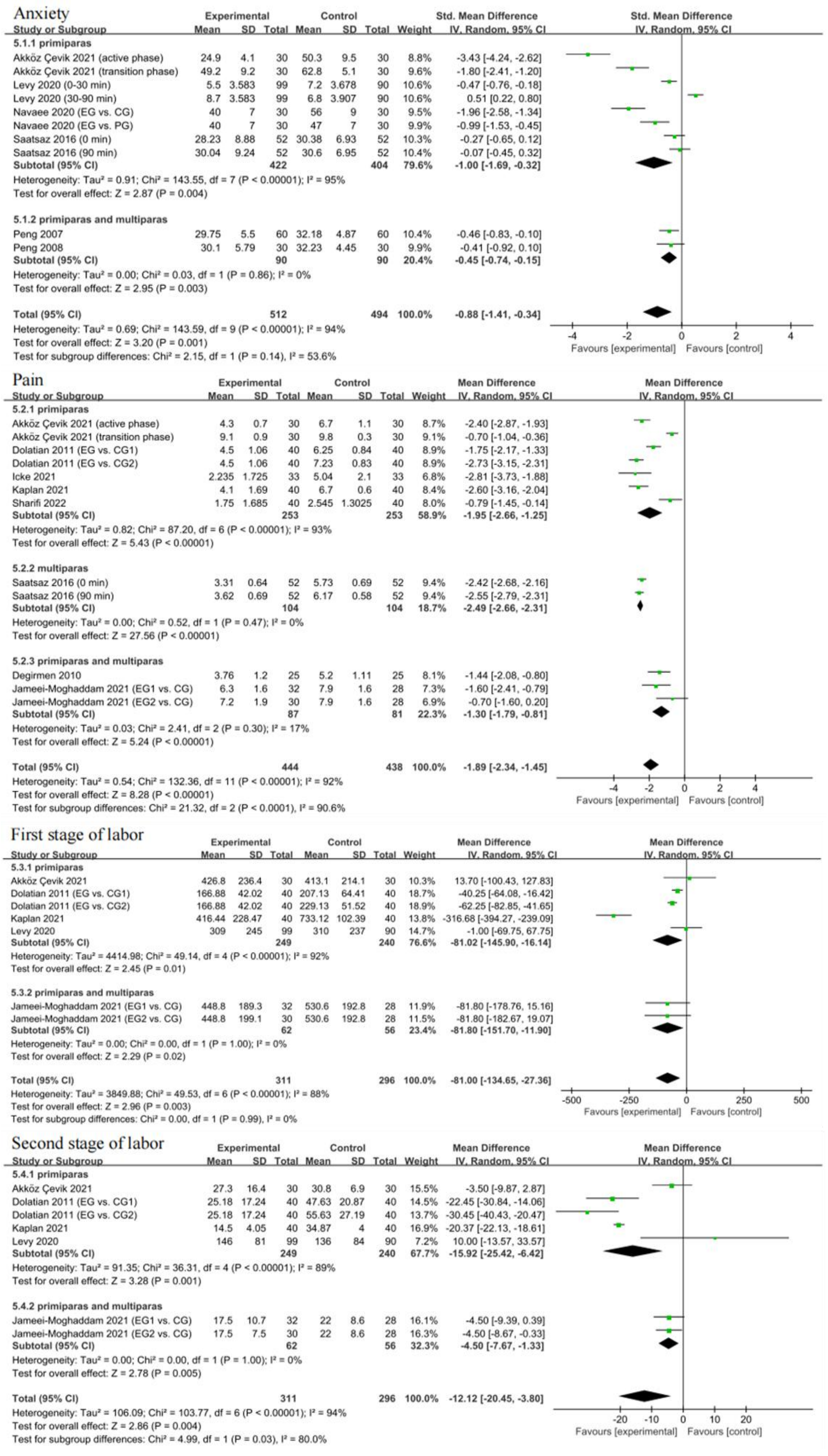

Figure S3. Subgroup analyses for anxiety, pain, first stage of labor, and second stage of labor, according to the times of pregnancies.

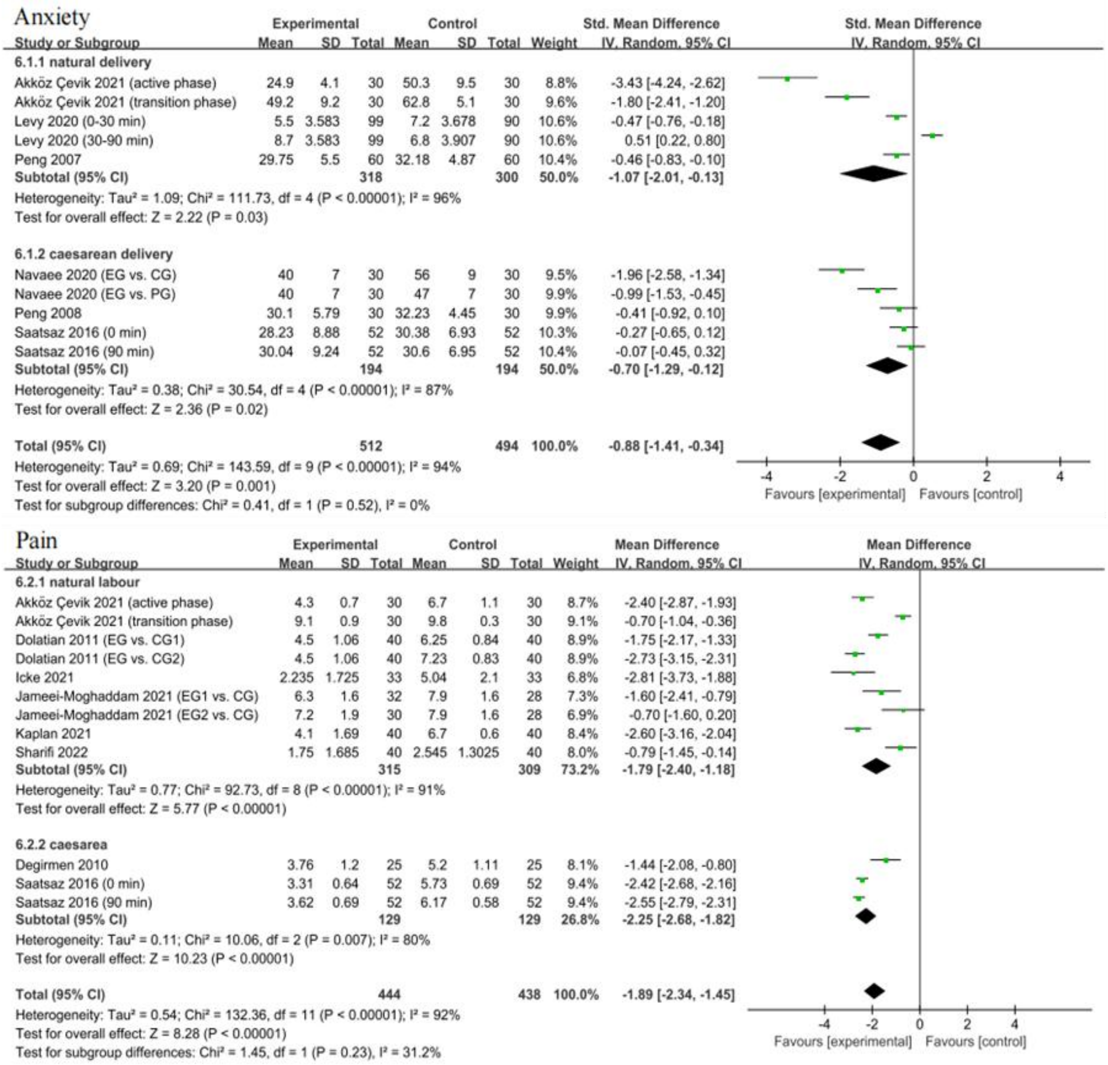

Figure S4. Subgroup analyses for anxiety and pain, according to the mode of delivery.

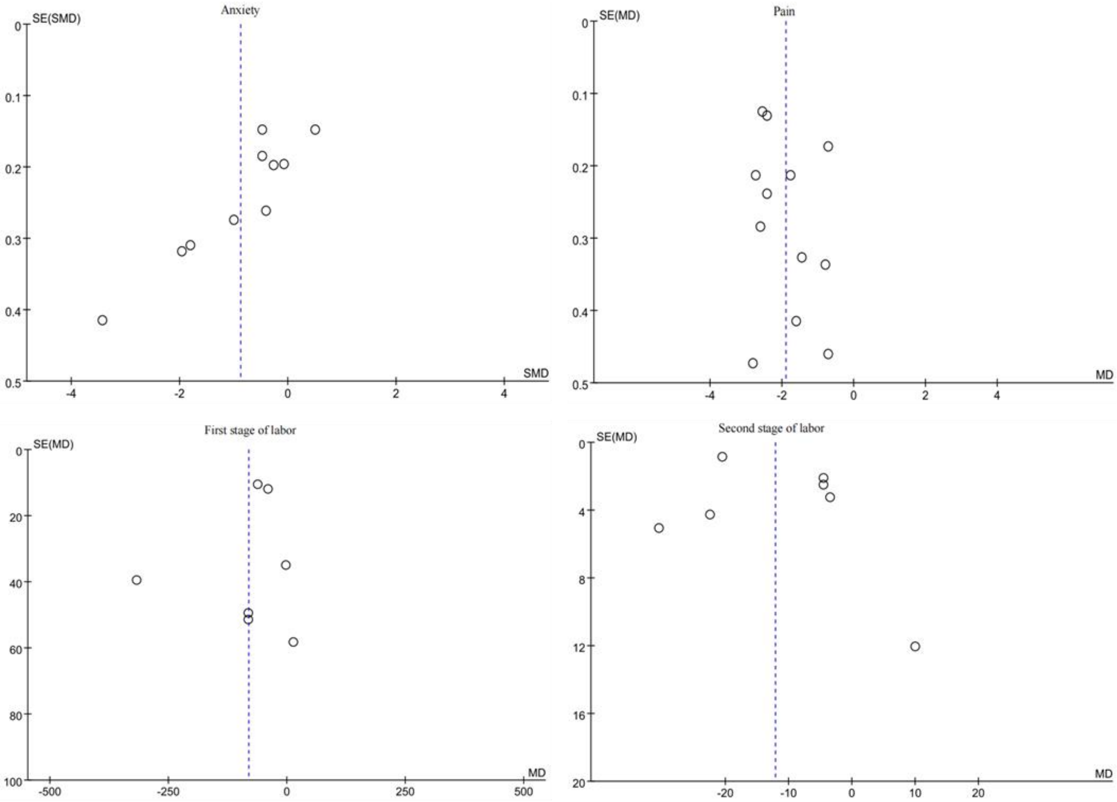

Figure S5. Funnel plots of anxiety, pain, first stage of labor and second stage of labor.

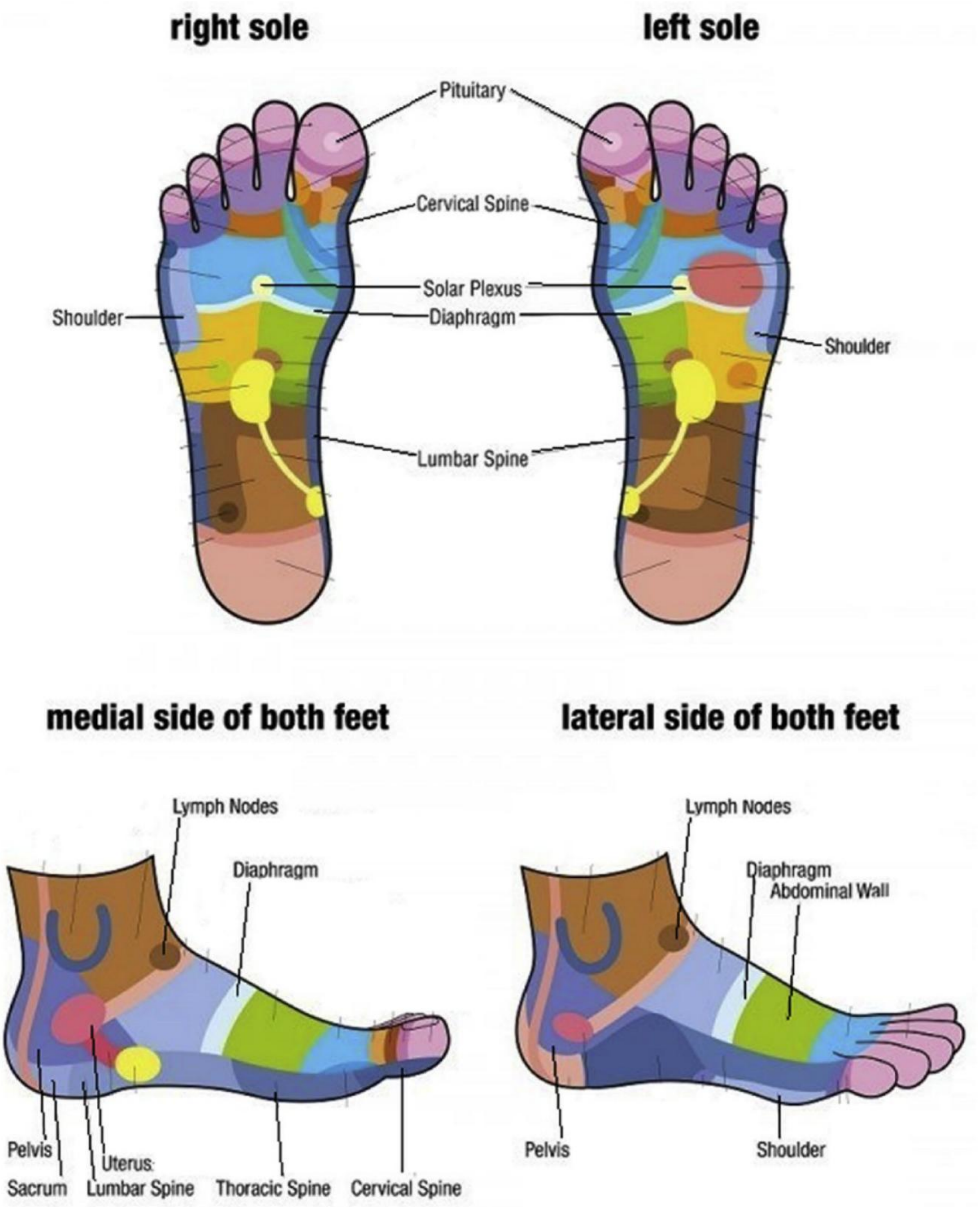

Figure S6. Schematic diagram of the reflex zones of the foot[21].
